# Decontamination of actual radioactive wastewater containing ^137^Cs using bentonite as a natural adsorbent: equilibrium, kinetics, and thermodynamic studies

**DOI:** 10.1038/s41598-022-18202-y

**Published:** 2022-08-16

**Authors:** Wasan A. Muslim, Talib M. Albayati, Salam K. Al-Nasri

**Affiliations:** 1 Iraqi Geological Survey/Ministry of Industry and Minerals, Baghdad, Iraq; 2 Department of Chemical Engineering, University of Technology-Iraq, 52 Alsinaa St., PO Box 35010, Baghdad, Iraq; 3 Radiation and Nuclear Safety Directorate Al-Tuwaitha Nuclear Site/Atomic Energy Authority, Baghdad, Iraq

**Keywords:** Environmental sciences, Engineering

## Abstract

Batch adsorption treatment using Iraqi bentonite as a natural adsorbent was adopted in this study to decontaminate actual ^137^Cs radioactive wastewater from the Al-Tuwaitha Nuclear Research Center, located south of Baghdad. The bentonite characterization was applied before and after treatment, using chemical compositions analyses, X-ray diffraction (XRD), scanning electron microscopy (SEM), energy-dispersive X-ray spectroscopy (EDX), Brunauer–Emmett–Teller (BET) surface area analysis and Fourier-transform infrared spectroscopy (FT-IR). The batch adsorption mode was applied with the initial radioactivity concentration (1440.5 Bq/L), solid/liquid ratio (1 g/L), pH (6–8), contact time (1.5 h), and temperature (298°K). The adsorption experiments showed a decontamination removal efficiency of about 95.66% of ^137^Cs. A Freundlich adsorption isotherm model was approved for the adsorption of ^137^Cs, with a coefficient of determination R^2^ = 0.998. A pseudo-second-order model fitted well with the adsorption of ^137^Cs, with R^2^ = 0.983. The positive value of ΔH° in the thermodynamic results indicated that the adsorption process was endothermic physisorption (ΔH° = 15.01 kJ mol^−1^), spontaneous and favorable (ΔG° = −7.66 kJ mol^−1^ K^−1^), with a very low degree of disorder (ΔS° = 0.076 kJ mol^−1^ K^−1^).

## Introduction

Radioactive wastewater is one of the most potentially dangerous pollutants that is generated from nuclear energy stations, medical nuclear applications, and various extractive industries worldwide. This radiation risks devastating the environment and living organisms because it is easily absorbed by soils and living creatures^1^. Since 1991, a great amount of liquid waste polluted with cesium (^137^Cs) has accumulated underneath the Al-Tuwaitha Nuclear Research Center, located near the city of Baghdad, Iraq^2^. ^137^Cs causes significant concern due to its gamma radiation, high solubility in liquid, half-life of 30 years, and its ability to cause serious harm to all living organisms. Accordingly, ^137^Cs contamination must be reduced to a safe limit to avoid the impacts of any leakage, both natural and industrial. The radioactivity of ^137^Cs (a long-lived radionuclide) wastewater can be minimized by selecting appropriate remediation. Various remediation methods have been utilized for the decontamination of ^137^Cs, such as evaporation, chemical precipitation, adsorption, solvent extraction, and filtration^3^. To remediate isotope substances in wastewater, adsorption is one of the most efficient methods, which is also simple in application and low in cost^4–6^. Bentonite is the most suitable clay used as a buffer and backfill material that is part of a significant group of natural materials mostly composed of fine-grained particles of minerals^7,8^. It is estimated to protect the surrounding environment from contamination with radionuclides due to its high swelling capacity and ability to adsorb cations on its negatively charged surface^9–11^. Various studies have compared bentonite and other clay minerals (e.g., illite, vermiculite, kaolinite, and palygorskite) on their adsorption efficiency, adsorption capacity, kinetics, isotherms, and parameters’ effects on the adsorption of cesium^12–14^. Additionally, the comparison studies involved modified and pillared bentonite. Moreover, bentonite is used with other materials as an adsorbent in many investigations, including zeolites, activated carbon, and biosorbents^15–17^. Briefly, in all cases (i.e., raw, modified, pillared, and hybrid), bentonite has displayed excellent adsorption of ^137^Cs such that for availability, inexpensive cost, and simple application, raw bentonite can be employed without any physical or chemical pretreatment^18^.

In this study, there are two main features that distinguish it from other similar ones. The first of these is that the Cs-137 radioactive liquid used in the research is different due to the properties it contains. The second is that the natural bentonite used is specific to a site. Iraqi raw bentonite was investigated as an adsorbent for the uptake of ^137^Cs from contaminated liquid at the Al-Tuwaitha site. Bentonite was characterized using chemical composition analyses, X-ray diffraction (XRD), energy-dispersive X-ray spectroscopy (EDX), scanning electron microscopy (SEM), particle size distribution, surface area, and Fourier-transform infrared (FT-IR) spectroscopy. Investigations studied the removal efficiency% of the effects of adsorption parameters, such as the adsorbent/liquid ratio, pH, sorbate concentration, contact time, and temperature. Additionally, evaluations were conducted of the influence of the adsorption capacity on the kinetic mechanisms, isotherm models, and thermodynamics.

## Materials and methods

### Sorbate preparation

**Figure 1:**
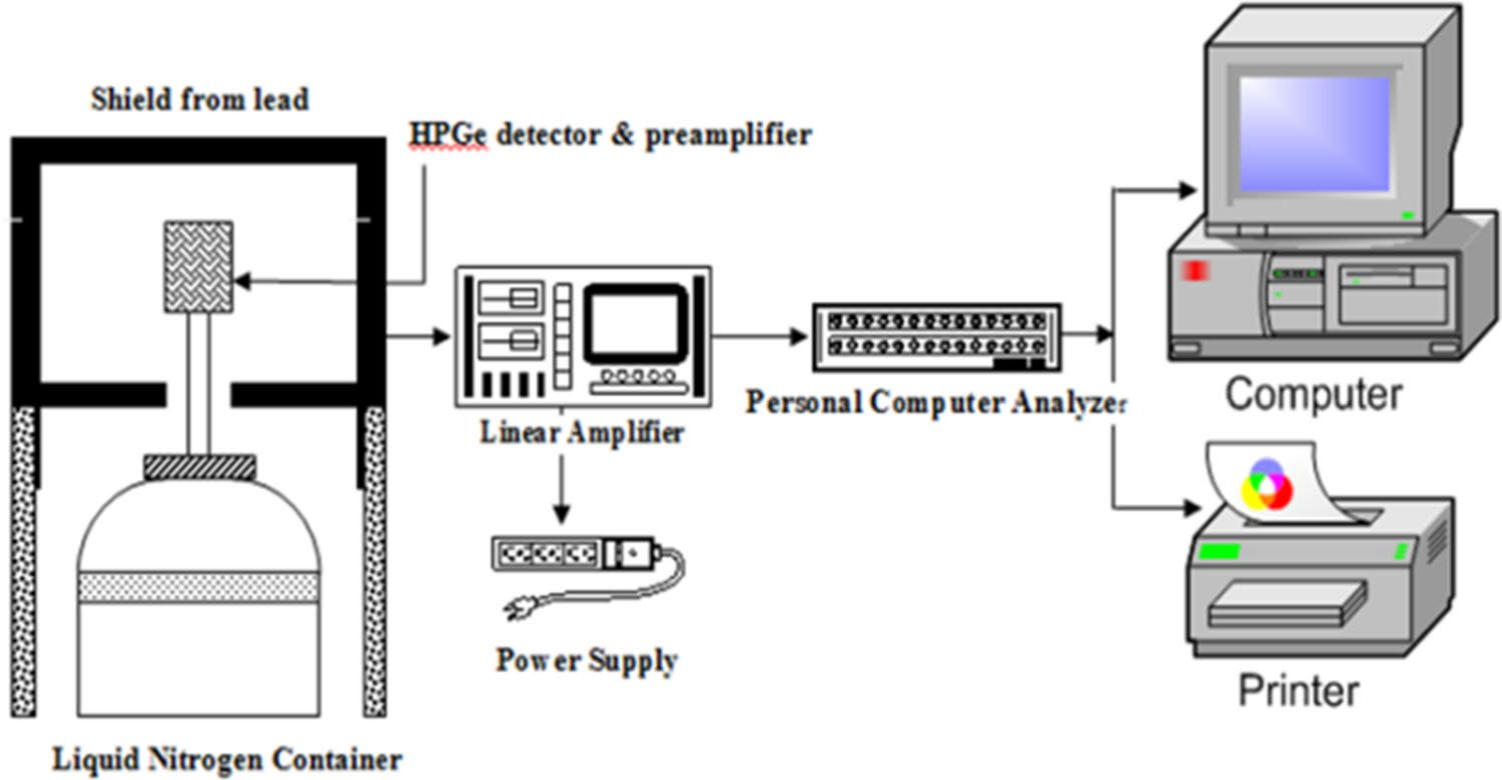
Gamma spectrometry technique of HPGe detector.

The source of the radioactive waste of Cs-137 is back to the previous activities of the Al-Tuwaitha nuclear site which was attacked during the Gulf war in 1990 and 2003. This radioactive wastewater contains in addition to the ^137^Cs traces of different types of short half-life radionuclides. All samples were measured and analyzed using gamma spectrometry analysis system with a closed end, coaxial, p-type model GEM65P4-95/ORTEC as shown in Fig. 1, which consists of a 65% relative efficiency of High purity germanium detector (HPGe), with the advanced gamma vision software. The energy and efficiency of the measuring system were calibrated using a multi-energy measuring source (MGS5.1045), using special containers for sec 3600. The dominant radionuclide of (^137^Cs) was measured at the energy of 661 keV. The specific activity of ^137^Cs for the stock liquid was 4.5 MBq/L, which was calculated using the below equation^19^:

$${\mathrm{Specific\, Activity}}\left(\frac{{\mathrm{Bq}}}{{L}}\right)=\frac{\begin{array}{c}\\ \left(\frac{{\mathrm{Area}}-{\mathrm{B.G}}}{{\mathrm{t}}}\right)\end{array}}{{\mathrm{I}}\gamma\%.{\mathrm{Eff}}\%.{\mathrm{V}}}$$

where A: specific activity in units (Bq/L). B.G: Back ground. V: the volume of the model. eff%: The percentage of efficiency. ${\mathrm{I}}\gamma \%$ : Intensity of gamma rays. t: the measurement time.

The contaminated liquid was diluted to a safe limit of about 7201.8 Bq/L. Analytical solutions of HCl and NaOH were used to regulate the pH of the ^137^Cs solutions for batch experiments using a gamma spectroscopy system HPGe detector. The total samples numbers are 25, and the experiment was repeated two times for each sample.

### Sorbent preparation and characterization

A bentonite clay sample was supplied from a deposit in Wadi Bashira of the Western Desert (Iraq). The clay sample was crushed by a jaw crusher (Retsch BB 1, Germany), then milled in a rotating cylinder ball mill and sieved in a 75-micron sieve opening. Bentonite clay was used without any physical or chemical pretreatment. Chemical wet analyses were carried out on bentonite to identify the composition of the contents by the Iraqi Geological Survey Laboratories. Mineralogical analyses were inspected using the Ital structure model MPD 3000. A MIRA3 TESCAN high-resolution analytical scanning electron microscope (SEM) with EDX was used to investigate the bentonite’s morphology. The particle size distribution analyses were accomplished by Brookhaven Instruments. The specific surface area (SSA) and the cation exchange capacity (CEC) for bentonite were obtained from technical reports of the Iraqi Geological Survey^20^. FT-IR analysis was conducted by ultraviolet and visible absorption spectrophotometer by a Bomem MB-Series FT-IR spectrometer according to ASTM E 1252–98.

### Adsorption procedure

The batch adsorption mode was applied using 50 ml of ^137^Cs solutions with different radioactive concentrations (1440.4–7201.8 Bq/L) and five pH values (i.e., 2, 4, 6, 8, and 10). Bentonite was added with various solid/liquid (S/L) ratios (0.5–2.5 g/L) in glass containers. The mixtures were combined using a shaking water bath at 200 rpm and at different bath temperatures (25–50 °C). At the end of the mixing times (which varied from 0.5 to 3 h), the samples were filtered using a 0.45-micron Whatman™ filter, and the radioactivity concentrations (specific activity Bq/kg) of ^137^Cs in the filtrates were assayed by an HPGe detector. The concentrations of ^137^Cs (µg/L) in the filtrates were estimated using Eqs. (1)–(4)^21^.

1 $$\mathrm{Specific\, Activity}\, \left({\mathrm{S.A.}}\right)=\frac{ \lambda \times Aav \times w}{M}$$

2 $${\mathrm{Rearrange}}\, w=\frac{ S.A. \times M}{ \lambda \times Aav}$$

where λ is the radioisotope decay constant (s^−1^), Aav is the Avogadro’s number (6.02 × 10^23^ nuclei/mol), w is the weight (g), and M is the atomic weight (g/mol).

3 $$\lambda =\frac{{\ln}2}{half\, life}= \frac{0.693}{{t}^{1/2}}$$

4 $${\mathrm{Concentration\, of\, Cs\, isotope\, in\, fltrate}}\, ({\mathrm{C}}) =\frac{w}{v}$$

where $v$ is the volume of sample (L).

The removal efficiency (R%) and adsorption capacity (q_e_) in (mg/g), were determined using Eqs. (5) and (6), respectively^22,23^:

5 $$R\%=\frac{concentration\, of\, adsorbed\, cesium}{initial\, concentration\, of\, cesium } =\frac{(C_{\rm o} - C_{\rm e} )}{C_{\rm o}} \times 100$$

6 $$q_{e}=\frac{amount\, of\, cesium\, adsorbed}{amount\, of\, adsorbent}=\frac{(C_{\rm o} - C_{\rm e})\times V}{M}$$

where C_o_ and C_e_ are the initial and equilibrium concentrations of radioactive cesium (Bq/L), respectively; V is the volume of the solution (L); and M is the weight of the clay mineral materials (g).

### Adsorption isotherm

Various types of adsorption isotherms models are mentioned in the literature, and the most remarkable ones have proceeded as linearized isotherm forms of either the Langmuir (Eq. 7), Freundlich (Eq. 8), Temkin (Eq. 9), or Hill (Eq. 10) models^24^. The adsorption isotherm rule at equilibrium presents the relationship between the adsorbent concentration and ion concentration in solution as follows:

7 $$\frac{C_{\rm e}}{q_{\rm e}}=\frac{1}{b\, q_{\max}}+\frac{C_{\rm e}}{q_{\max}}$$

where C_e_ is the activity concentration of cesium alone in solute (mg/L), q_e_ is the adsorbed material on the adsorbent surface (mg/g), q_max_ is the maximum monolayer capacity (mg/g), and b is the Langmuir constant (L/g).

8 $$Log\, q_{\rm e} =Log\, K_{\rm f}+\frac{1}{n} Log\, C_{\rm e}$$

where K_f_ is the Freundlich constant (mg/g) (which approximately indicates the adsorption capacity), and 1/n is the intensity of adsorption (which evaluates the adsorption process).

9 $$q_{\rm e}= \frac{RT}{b} Ln\, C_{\rm e}+ \frac{RT}{b} Ln\, A$$

where b is the isotherm Temkin constant (which is related to the adsorption temperature), A is the binding constant of the equilibrium (L/g), R is the ideal gas constant (8.314 j/mol. K), and T is the absolute temperature (°K).

10 $$Log \frac{q_{\rm e}}{{q}_{sh}-q_{\rm e}}=n_{\rm h}Log\, C-Log\,{K}_{D}$$

where *n*_h_ Hill cooperativity coefficient of the binding interaction, ${q}_{sh}$ and K_D_ Hill isotherm constant. Furthermore, in this model if *n*_h_ =1 that means the binding is hyperbolic or non-cooperative, if *n*_h_ > 1, binding has positive cooperativity, while negative cooperativity occurs when *n*_h_ < 1.

### Adsorption kinetics

The mechanism of ^137^Cs adsorption on bentonite surfaces was evaluated by using the data resulting from the effect of contact time. The evaluation is based on the application of three kinetic adsorption linearized models: pseudo-first-order (Lagergren model), pseudo-second-order (Ho model), and intraparticle diffusion (Weber-Morris model), which are represented by Eqs. (11), (12), and (13), respectively^25^.

11 $$ln(q_{\rm e} - q_{\rm t})=lnq_{\rm e}-k_{1}t$$

12 $$\frac{t}{q_{\rm t}} =\frac{1}{k_{2}q_{\rm e}^{2}}+ \frac{t}{q_{\rm e}}$$

13 $$q_{\rm t}k_{\rm p}\, {t}^{1/2} + C$$

where q_t_ and q_e_ are the adsorption capacity (mg/g) at time t (min) and at equilibrium, respectively; K_1_ and K_2_ are pseudo-first order (min^−1^) and pseudo-second order (g/mg min) adsorption rate constants, respectively; K_P_ is the constant of the intraparticle diffusion rate (mg/g min^0.5^); and C is the constant of the intraparticle diffusion.

### Adsorption thermodynamics

Thermodynamic studies were conducted by evaluating the physicochemical properties and thermochemical nature of Cs adsorption onto bentonite. K_c_ values were estimated by Eq. (14) for different temperatures (i.e., 25, 35, 40, 45 and 50 °C), and different values of ΔG°. ΔH° and ΔS° values for these temperatures were estimated from equating Eqs. (15) and (16) to generate Eq. (17), which is called van’t Hoff model^26^.

14 $$Kc=\frac{q_e}{C_e}$$

15 $$\Delta G^\circ =-RT\, ln\, Kc$$

16 $$\Delta G^\circ =\Delta H^\circ - T\, \Delta S^\circ$$

17 $$Ln\, Kc=\frac{\Delta S^\circ }{R}-\frac{\Delta H^\circ }{RT}$$

where Kc is the equilibrium constant, R is the ideal gas constant (8.314 J/mol. K), T is the absolute temperature (°K), ΔG° is the adsorption Gibbs free energy change (kJ/mol), ΔH° is the adsorption free enthalpy change (kJ/mol), and ΔS° is the adsorption free entropy change (kJ/mol K).

## Results and discussion

### Sorbent characterizations

**Figure 2:**
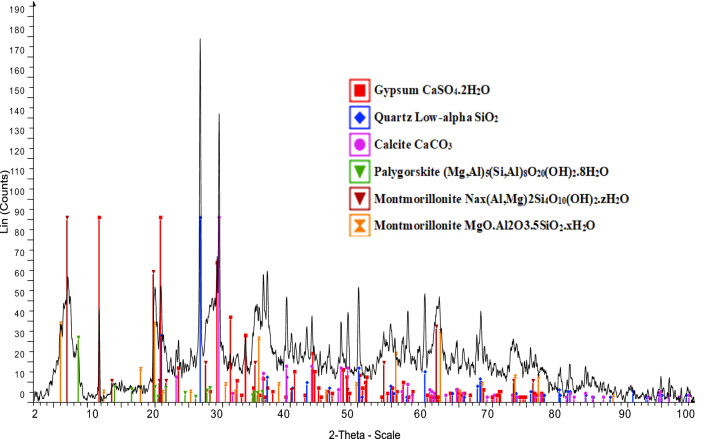
XRD pattern of Bentonite clay.

The results of the chemical and mineralogical analyses (shown in Table 1 and Fig. 2) indicated that the applied bentonite type was low-grade bentonite ore. There was a high content of impurities of major minerals (e.g., silica, calcite, and gypsum) as displayed in Fig. 2, while the clay minerals, (Ca, Mg and Na montmorillonites) were present as minor minerals. The bentonite had not been previously purified or modified, which is clearly shown in these results.

**Table 1:** Chemical analyses of Bentonite clay ore.

Chemical compositions	SiO_2_	Al_2_O_3_	Fe_2_O_3_	CaO	MgO	SO_3_	L.O	Na_2_O	K_2_O	Cl
%	48.16	15.46	6.46	8.10	3.56	2.42	13.03	1.12	0.47	0.71

**Figure 3:**
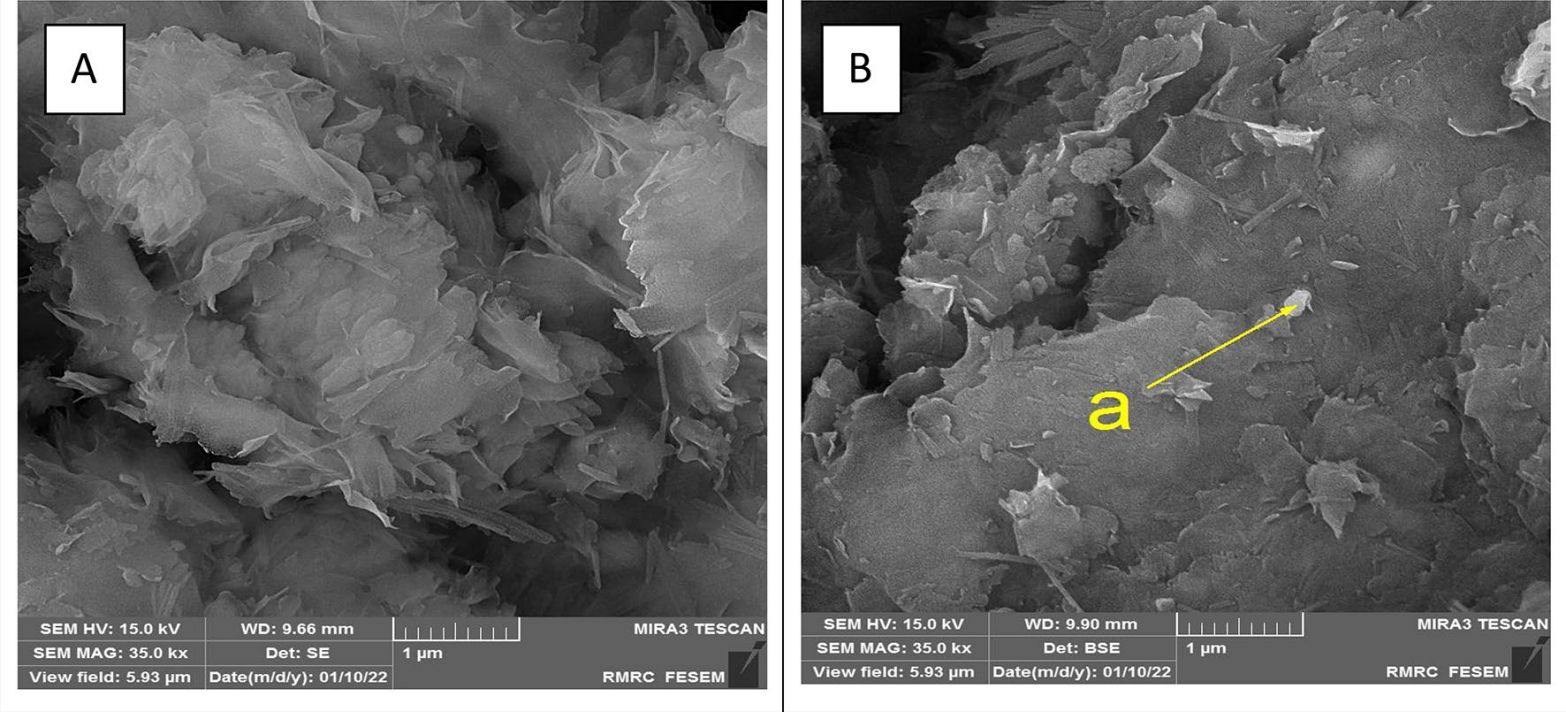
SEM image for Bentonite clay (**A**) Before adsorption (**B**) After adsorption.

Scanning electron microscopy (SEM) images for bentonite are shown in Fig. 3. The SEM showed the morphology of the bentonite structure before adsorption, as shown in Fig. 3a, with a fine and close plate-like shape structure. In contrast, after adsorption (Fig. 3b), the layered structure was disordered, and the particles were almost flat.

**Figure 4:**
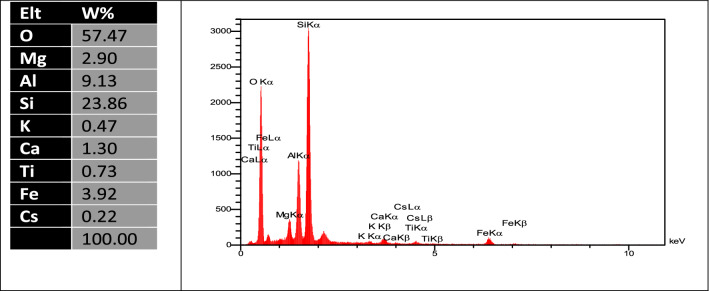
EDAX analysis of Bentonite after adsorption.

Energy-dispersive x-ray analysis (EDX) was achieved at point (a) on the bentonite surface after adsorption, as shown in Fig. 3b. The EDX spectrum detects the presence of ^137^Cs by Lα and Lβ in the range of 4–5 keV (see Fig. 4). The quantitative analysis at the same point indicates the main composition percentage of bentonite clay and the loaded ^137^Cs.

**Figure 5:**
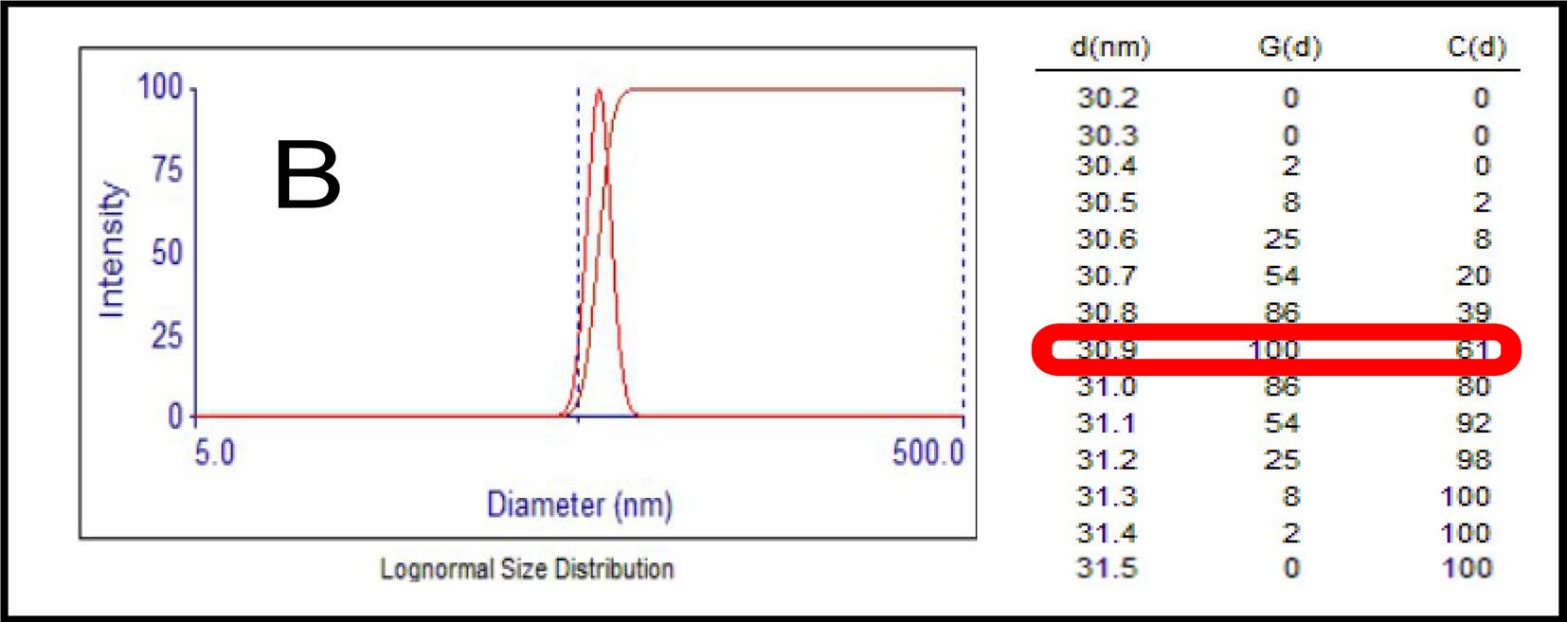
The particle size distribution of Bentonite clay.

The particle size of bentonite was investigated using a Brookhaven NanoBrook 90Plus particle size analyzer, which uses dynamic light scattering (DLS) principles. The mean particle size of the bentonite is presented in Fig. 5. It was found that the 30.9 nm particle had the highest intensity value, with the specific surface area of bentonite listed in Table 2.

**Table 2:** The mean particle size with a specific surface area and cation exchange capacity for Bentonite.

Sample	Mean Particle Diameter (nm)	St. Deviation	Density (g/cm^3^)	Specific Surface Area (SSA) m^2^/g	CEC g/100 g
Bentonite	30.9	1.07	2.5	77.7	25.6

**Figure 6:**
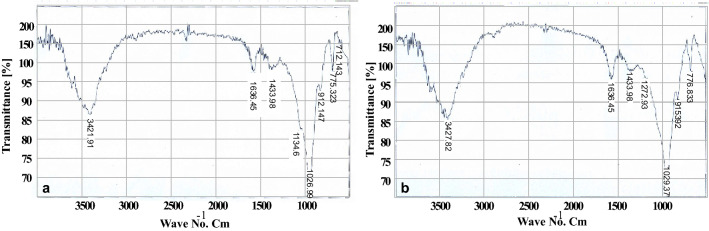
FTIR Bentonite clay (**a**) before adsorption (**b**) after adsorption.

Montmorillonite (smectite) has a 2:1-layer structure consisting of an octahedral alumina sheet sandwiched between two opposing tetrahedral silica sheets. FT-IR spectra of bentonite before and after the cesium adsorption process were measured to investigate the structural changes. However, the spectrum of bentonite before the adsorption in Fig. 6b showed the following bands: a stretching vibration Al–OH/OH, bending vibration H–OH, stretching vibration Si–O–Si, and a stretching vibration Al–OH–Al at 3421, 1636, 1026/776, and 912 cm^−1^, respectively. Figure 6a shows the spectrum after the cesium adsorption, with a slight shift towards a higher frequency on the Al–OH–Al band than in the raw bentonite (from 3421 to 3427 cm^−1^)^27^.

### Batch adsorption parameters

#### Effect of the solid/liquid ratio on adsorption

**Figure 7:**
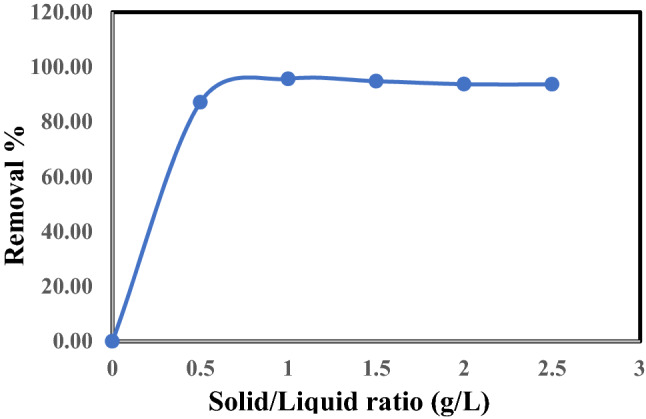
Effect of solid/liquid on the removal of Cs^137^ by Bentonite at room temperature, t = 1 h., Co = 0.5 μg/L and pH = 6.

The effect of the solid/liquid (S/L) ratio on the adsorption of ^137^Cs is shown in Fig. 7. The bentonite dosage was 0.5, 1, 1.5, 2, and 2.5 g/L of contaminated liquid with all other parameters remaining constant. The removal of cesium increased as the ratio of bentonite to liquid rose. The removal of ^137^Cs at bentonite amounts greater than 1 g/L remained almost constant. Thus, the equilibrium (1 g/L) ratio was considered to be the optimal dose, with an adsorption removal of 95.66%. Wu et al. (2009)^26^ studied the adsorption rate of Cs^+^ by changing the liquid/solid ratio from 1000:1 mL/g to 50:1 mL/g, which decreased the adsorption rate from 63.5 to 98.0%. This can be explained by the fact that the odds of Cs^+^ colliding on the surface of the montmorillonite increased with additional adsorbent active sites. The increase in adsorbent amounts meant an increase in the surface area and the number of active sites of adsorbent surface exposed for ion exchange, as found by Abou-Lilah et al. (2020)^27^.

#### Effect of pH on adsorption

**Figure 8:**
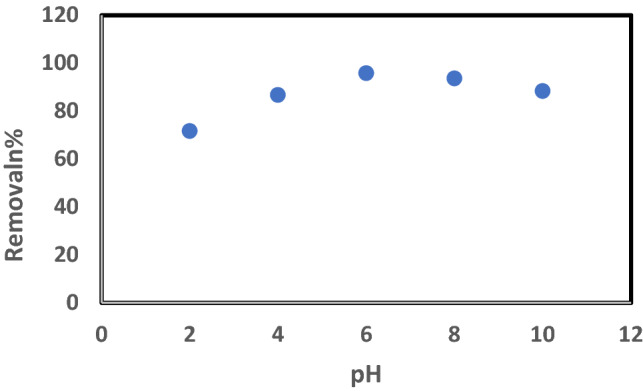
Effect of initial pH on the removal of Cs^137^ by Bentonite at room temperature, t = 1 h., Co = 0.5 μg/L and solid/liquid = 1.5 g/L.

The investigation of the initial pH effect on the ^137^Cs adsorption removal by bentonite is depicted in Fig. 8. The uptake of ^137^Cs from wastewater occurred at higher pH levels (6–7), while it decreased as the acidity increased from 6 to 4 and the alkalinity increased from 8 to 10. This result can be explained due to the negative electrostatic charge of the natural bentonite clay sheet surfaces, which attracts the positive charge of the ^137^Cs to its surface to encourage ion exchange. When the acidity increased, the H^+^ ions increased, competing with the ^137^Cs ions on the surface of the clay. On the other hand, the high alkalinity creates cesium complexes, such as Cs (OH_2_)^−^, which are repulsed by the surface and force the cesium to desorption. This result agrees with the conclusion of the pH investigation by Galambos et al. (2010), which indicated that the adsorption percentage approaching 97% was reached at pH levels between 6 and 8. Therefore, for maximum Cs-adsorption, the disposal of high-level radioactive wastewater should be carried out at a pH of 7^28^.

#### Effect of contact time and adsorption kinetics

**Figure 9:**
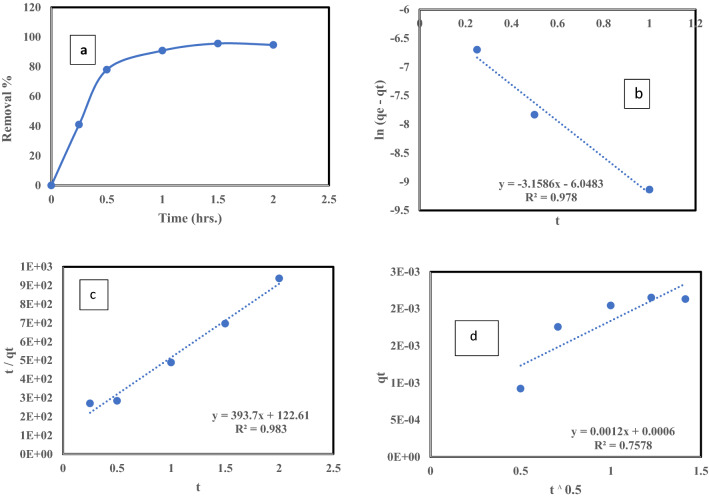
(**a**) Effect of contact time on the removal of Cs^137^ (**b**) pseudo first-order adsorption kinetic model (**c**) pseudo second-order adsorption kinetic model d: intra-particle diffusion adsorption kinetic model by Bentonite at room temperature, pH = 6, Co = 0.5 μg/L and solid/liquid = 1.5 g/L.

The influence of contact time on the ^137^Cs uptake percent from wastewater using bentonite is plotted in Fig. 9a. The adsorption quickly increased in the first 30 min and reached equilibrium at 1.5 h, with a removal % of 95.66%. The effect of contact time on the ^137^Cs adsorption using raw bentonites has been studied^29,30^. The faster ^137^Cs uptake percent was associated with the initial presence of many empty active sites for adsorption on the clay surfaces. Then, with increasing contact time, the uptake percent gradually increased because most active sites were already occupied and remained in that condition until equilibrium was reached, when all active site became vacant^31,32^. Kinetic models were employed to simulate the experimental data in the contact time study in relation to the adsorption capacity (q_t_). The linear forms of the pseudo-first-order model (Eq. 11), pseudo-second-order model (Eq. 12), and intraparticle diffusion model (Eq. 13) are plotted in Fig. 9b–d, respectively. For further discussion, the kinetic characteristics were estimated from the slopes and intercepts of these linear models and tabulated in Table 2. The pseudo-first and second-order models have a good fit to the ^137^Cs adsorption mechanism, with high regression coefficients (R^2^) of 0.983 and 0.978, respectively. The theoretical amount of ^137^Cs adsorbed at equilibrium q_e_ suggested by the pseudo-first-order model was about 2.36 × 10^−6^ mg/g, which was closer to the experimental q_e_ of about 2.15 × 10^−6^ mg/g than that of the pseudo-second-order model, which was approximately 2.54 × 10^−6^ mg/g, as shown in Table 3. Although there is only a small difference in the q_e_ between the first- and second-order models, the regression coefficient R^2^ best fits the second-order models.

**Table 3:** Kinetic models parameters for the adsorption of Cs^137^ by Bentonite at room temperature, pH = 6, Co = 0.5 μg/L and solid/liquid = 1.5 g/L.

Pseudo-First Order model			Pseudo-second Order model			Intra-particle diffusion model		
*K*_1_ (min^−1^)	q_e_ (mg/g)	R^2^	K_2_ (g/mg min)	q_e_ (mg/g)	R^2^	K_p_ (mg/g.hr_._^0.5^)	C (mg/g)	R^2^
0.052	2.36E-6	0.978	21,069.5	2.54E-6	0.983	0.0012	0.0006	0.757

The intraparticle diffusion model does not fit with the experimental data because the regression coefficient R^2^ had a low value (0.757), indicating poor diffusion and that adsorption mainly occurred on the surface. The kinetics of ^137^Cs of adsorption fitted with the pseudo-second-order model, meaning that the controlling mechanism step was ion-exchange by electrostatic interactions, which played a key role. Most of the previous studies on ion adsorption on clays were represented by the pseudo-second-order kinetic model^32–34^. Film diffusion can be considered the rate-controlling step if the adsorption process is marked by inefficient mixing, small solute size, and low concentration; otherwise, intraparticle diffusion controls the process.

#### Effect of ^137^Cs initial concentration and adsorption isotherm

**Figure 10:**
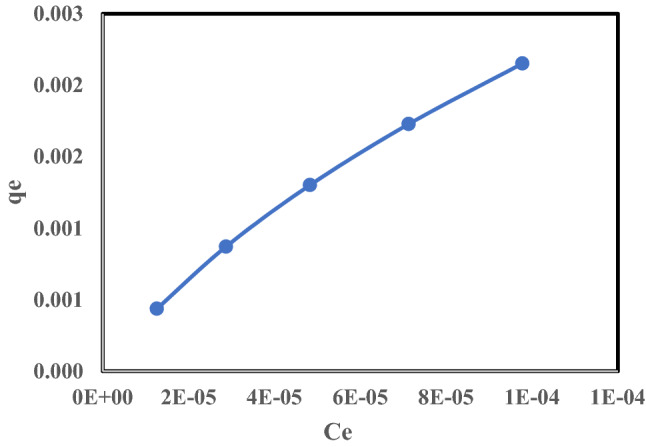
Effect of Cs^137^ concentrations on the amount of Cs^+^ ions adsorbed.

**Figure 11:**
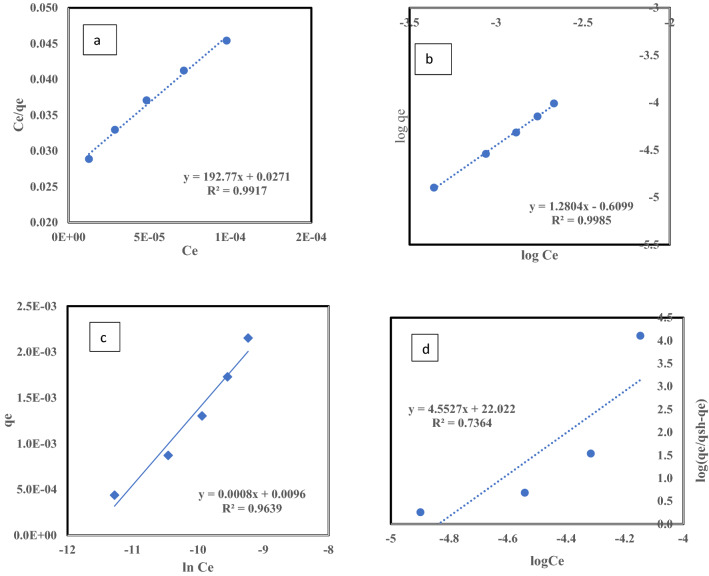
(**a**) Langmuir adsorption isotherm (**b**) Freundlich adsorption isotherm (**c**) Temkin adsorption isotherm (**d**) Hill adsorption isotherm .by Bentonite at room temperature, pH = 6, t = 1 h., and solid/liquid = 1.5 g/L.

This study investigated the effects of the initial concentrations of adsorbate on the amount of Cs^+^ ions sorbed onto the surface of the clay, finding that increasing the initial adsorbate concentrations caused an increase in the equilibrium concentration. Thus, the amount of ^137^Cs isotope sorbed onto the surface of the clay increased, as illustrated in Fig. 10. The adsorption isotherm illustrates the adsorption situation at equilibrium and reveals the influence of the adsorbate ion concentration in the bulk solution on the total adsorbed amount. Figure 11a–d clarify the application of the linear form of either the Langmuir (Eq. 7), Freundlich (Eq. 8), or Temkin (Eq. 9), Hill (Eq. 10) adsorption isotherms of ^137^Cs. To verify the experimental data, the correlation coefficient parameters of these isotherms was estimated from the slopes and intercepts of linearization, as shown in Table 4. From the obtained correlation coefficient (R^2^) values for these models, it was evident that the isotherm model’s applicability for cesium removal using raw bentonite follows this order: Freundlich > Langmuir > Temkin > Hill, as shown in Table 4. The R^2^ value was greater than 0.99 for both the Langmuir and Freundlich isotherm models, making them more suitable to elucidate the adsorption isotherm of the adsorbed ions than the Temkin model, with R^2^ = 0.963. While Hill model with n_h_ = 4.5 > 1 indicate that positive cooperative adsorption, this mean influencing of other binding sites on the same adsorbent, but the data did not fit with Hills model at R^2^ = 0.736^35^. Depending on the comparison between the correlation coefficient parameters of the isotherm models, for the Langmuir model, the maximum monolayer adsorption capacity q_max_ was 5.18E-6 mg/g and R_L_ (the dimensionless equilibrium parameter value) was close to 0.058. An R_L_ value in the range from 0–1 indicates that the adsorption process is favorable. However, in the Freundlich model, the adsorption intensity constant (n) was 1.28, which indicates moderate adsorption, whereas, for good adsorption, the (n) value must range from 2 to 10^36^.

**Table 4:** The correlation coefficient parameters of isotherms for the adsorption of Cs^137^ by Bentonite at room temperature, pH = 6, t = 1.5 h. and solid/liquid = 1.5 g/L.

Langmuir			Freundlich			Temkin			Hill		
*q*_max_ (mg/g)	R_L_	R^2^	K_f_ (mg/g)	n	R^2^	b (kJ/mol)	A (L/g)	R^2^	n_h_	K_D_	R^2^
5.18E−6	0.058	0.991	0.0016	1.28	0.998	3096	162,754.7	0.963	4.553	2.72E-10	0.736

These favorable characteristics support the validity of using the Langmuir adsorption isotherm model. The adsorption by raw bentonite is monolayered sorption predominantly on distinct localized adsorption sites, with no transmigration of the adsorbate in the plane of the surfaces and assumes uniform energies of monolayer sorption onto the adsorbent surface, which was also concluded for the adsorption by raw bentonite by Abou-Lailah et al. (2020)^27^ and Balouch et al. (2013)^37^.

#### Effect of temperature and adsorption thermodynamics

**Figure 12:**
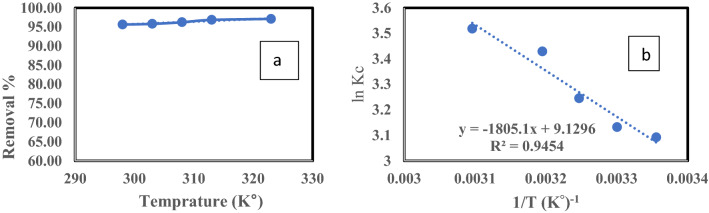
(**a**) Effect of Temperature on the removal of Cs^137^. (**b**) (Van’t Hoff) relation adsorption by Bentonite, pH = 6, Co = 0.5 μg/L, t = 1 h. and solid/liquid = 1.5 g/L.

The effect of temperature on ^137^Cs adsorption is shown in Fig. 12a, which indicates that ^137^Cs removal increased slightly with the increasing temperature of the adsorption process from 298–323°K. Gibbs free energy (ΔG°), enthalpy (ΔH°), and entropy (ΔS°) provide an evaluation of the adsorption process spontaneity, the adsorption process nature, and the applicability of the adsorbent, respectively. The equilibrium constant (Kc) was determined from Eq. (14), while ΔH° and ΔS° were evaluated from the slope and intercept of a graph of Ln K_c_ versus 1/T (van’t Hoff), as illustrated in Fig. 12b. The thermodynamic parameters for ^137^Cs adsorption are listed in Table 5.

**Table 5:** The correlation parameters of thermodynamic for the adsorption of Cs^137^ by Bentonite, pH = 6, t = 1 h. Co = 0.5 μg/L and solid/liquid = 1.5 g/L.

Temperature (K°)	LnK_c_	ΔG° (kJ/mol)	ΔH° (kJ/mol)	ΔS° (kJ/mol. K°)	R^2^
298	3.092	-7.66	15.01	0.076	0.945

The negative value of ΔG° confirms the spontaneous nature of the adsorption process. The values of ΔG° (−7.66 kJ/mol) indicated that the process of physisorption had occurred, as values of ΔG° for the physisorption process are generally between 20 and 0 kJ/mol. The positive value of ΔH° (15.01 kJ/mol) confirmed the endothermic nature of the process. The positive value of ΔS° (0.076 kJ/mol/°K) signified that randomness among the solid–liquid interfaces in the bulk solution had increased during adsorption equilibrium^38,39^. The numerical value of ΔH° supplies the type of sorption features, such that if the ΔH° value is from 2–21 kJ/mol, the adsorption process is physisorption, but if it ranges from 80–400 kJ/mol, it is chemisorption^40^. Based on the range of the ΔH° value, the ^137^Cs adsorption occurred using an ion-exchange mechanism^41^.

## Comparison study

The optimum equilibrated conditions resulting from the batch adsorption experiments were a solid:liquid ratio of 1 g/L, pH (6–8), initial cesium activity concentration (7201.8 Bq/L), contact time (1.5 h), and temperature (298°K). The adsorption efficiency achieved about 95.66% at the optimum condition. The results of this study were compared with some previous studies using the same approach. The batch adsorption conditions are listed in Table 6. The results of this study are similar to the adsorption efficiency of bentonite found in other studies^42^. Also, the Langmuir isotherm fit with an adsorption isotherm of ^137^Cs mentioned by^43^. It should be noted that the raw bentonite in this study was untreated or modified.

**Table 6:** The results comparison between the previous studies and this study.

Sorbent type	Removal efficiency (%)	Preferred isotherm model	Correlation coefficient (R^2^)	References
Chinese modified Bentonite	98	Langmuir	0.99	^27^
Slovak Bentonite and montomorilite	97	–	–	^28^
Egyptian Bentonite	93	Langmuir	0.98	^26^
Brazilian Bentonite	95.6	Langmuir and Freundlich	–	^44^
Iraqi Bentonite	95.66	Langmuir	0.99	Current study

## Conclusions

Iraqi raw bentonite without any pretreatments successfully decontaminated ^137^Cs from the Al-Tuwaitha radioactive wastewater by batch adsorption. The adsorption experiments indicated that about 95.66% of ^137^Cs was decontaminated under the following conditions: initial activity concentration (1440.5 Bq/L), solid:liquid ratio (1 g/L), pH (6–8), contact time (1.5 h), and temperature (298°K). The adsorption in this ^137^Cs isotherm study proved the validity of using the Langmuir adsorption isotherm model. The kinetics of the adsorption of ^137^Cs fitted with a pseudo-second-order model, meaning that the controlling mechanism step was ion-exchange by electrostatic interactions on the bentonite clay surface. The positive value of ΔH° in the thermodynamic study confirmed the endothermic nature of the process. The ΔH° range value indicated that ^137^Cs adsorption was physisorption and used an ion-exchange mechanism. These results support the notion that batch adsorption with raw bentonite is beneficial for several reasons, including its high efficiency, simple application, low cost, and use of eco-friendly material. Additionally, this study provides useful information about the adsorption process to develop a suitable continuous method for radioactive wastewater treatment. Finally, the adsorption technique minimizes the volume of one liter of radioactive wastewater to the volume of one gram of loaded bentonite, which can be easily sequestered in a landfill after suitable cementation.

## Data Availability

All data generated or analyzed during this study are included in this published article.
